# Culturally aware mentorship: Lasting impacts of a novel intervention on academic administrators and faculty

**DOI:** 10.1371/journal.pone.0236983

**Published:** 2020-08-07

**Authors:** Veronica Y. Womack, Christine V. Wood, Stephanie C. House, Sandra C. Quinn, Stephen B. Thomas, Richard McGee, Angela Byars-Winston

**Affiliations:** 1 Searle Center for Advancing Teaching and Learning, Northwestern University, Evanston, IL, United States of America; 2 Department of Medical Social Sciences, Northwestern University Feinberg School of Medicine, Chicago, IL, United States of America; 3 Institute for Clinical and Translational Research, University of Wisconsin–Madison, Madison, WI, United States of America; 4 Department of Family Science and Maryland Center for Health Equity, School of Public Health, University of Maryland, College Park, MD, United States of America; 5 Department of Health Policy and Management and Maryland Center for Health Equity, School of Public Health, University of Maryland, College Park, MD, United States of America; 6 Faculty Affairs, Northwestern University Feinberg School of Medicine, Chicago, IL, United States of America; 7 Department of Medicine, Center for Women’s Health Research, University of Wisconsin–Madison, Madison, WI, United States of America; King Fahd University of Petroleum & Minerals, SAUDI ARABIA

## Abstract

National efforts to address the diversity dilemma in Science, Technology, Engineering, and Math (STEM) often emphasize increasing numbers of historically underrepresented (HU) students and faculty, but fall short in instituting concrete changes for inclusion and belonging. Therefore, increasing the pool of senior faculty who wish to become guides and advocates for emerging scientists from HU populations is an essential step toward creating new pathways for their career advancement. As a step toward achieving this goal, we created a novel eight-hour intervention on Culturally Aware Mentoring (CAM), a program of the National Research Mentoring Network (NRMN) targeted to faculty and administrators. A previous report of surveys at the end of the CAM sessions revealed substantial awareness and knowledge gains, with participants expressing intentions to use and implement new skills they had learned. In this paper, we provide the results of our thematic analysis of qualitative interviews with academic administrators and faculty, 18–24 months after participation in CAM. Interviews were designed to determine: 1) What changes in self-perceptions and interactions occurred as a result of participation in CAM? 2) What specific components of CAM are associated with changes in individual beliefs and practices? 3) How did participants actively make changes after the CAM workshop? 4) What barriers or challenges do participants encounter after the CAM intervention? The results demonstrate the lasting influences of CAM on participants’ awareness of cultural differences, their assumptions about and approaches toward interactions with colleagues and students, and their efforts to change their behaviors to promote inclusive practices in their mentoring and teaching of HU students in STEM. Our findings provide evidence that CAM can be incorporated into existing mentor training programs designed to improve the confidence and capacity of senior research faculty mentors to make culturally-informed, scholar-centered decisions to more deliberately recognize and respond to cultural differences within their mentoring and collegial relationships.

## Introduction

The diversity dilemma within the biomedical sciences is well documented, and national initiatives to address the situation are well underway [1]. National leaders have prioritized increasing and improving the training and access of individuals from historically underrepresented (HU) groups in NIH-supported research areas, particularly American Indians/Alaska Natives, Black/African Americans, Hispanics/Latinos, and Hawaiians/Pacific Islanders [2]. However, success in recruitment of HU scholars exposes the importance of inclusion; in other words, belonging matters [3]. When the numbers of HU scientists increase within an institution, creating an inclusive scientific community in which all scientists feel welcomed and valued is essential. Writing in *Science Magazine* in 2017, Puritty et al. stated that without inclusion, diversity initiatives may not be enough [4]. Focusing on head counts may be an important metric but also insufficient to solve the diversity dilemma.

Despite wide-ranging efforts to attract and retain more individuals from HU racial/ethnic groups, sustained efforts to prepare existing faculty who mentor emerging scientists continues to be inadequate to meet growing demand. Existing faculty and administrators need a roadmap to navigate the social and cultural dynamics that come with a more demographically diverse student body. Whereas diversity-focused efforts may increase the numbers of diverse individuals pursuing biomedical sciences careers, a focus on inclusion necessitates addressing the lived experiences, treatment, and sense of belonging of HU individuals in specific scientific training environments. Increasing numbers of diverse trainees is not enough to counter the effects of the prevailing culture characterized by bias and prejudice related to race, ethnicity, gender, and other identities. One factor needed to solve the diversity dilemma in the biomedical sciences is a process for developing mentors who can effectively engage with and develop the talent of all individuals, particularly those from racial/ethnic groups historically underrepresented in these fields.

The purpose of this paper is to describe how participation in the implementation of a Culturally Aware Mentoring (CAM) intervention influenced the attitudes, beliefs, and behaviors of research mentors, staff, and administrators. Our previous report described the design of CAM and immediate reactions to it by participants [5]. In this paper, we used qualitative methods to investigate the lasting effects of CAM training on participants in terms of outcomes and perceived changes in mentoring and other academic relationships 18–24 months after the intervention. We report the results of a thematic analysis from interview data we collected from a convenience sample of participants (N = 24).

The day-long CAM intervention is comprised of three sections. In the first section, we focus on intrapersonal awareness and conduct exercises that encourage participants to reflect on their racial and ethnic identities. Multicultural [6, 7] and feminist theories [8, 9] posit that awareness of how one's social identity shapes worldviews and experiences can help individuals better understand their own perspectives and learn to interact with people from similar and different backgrounds. More pointedly, Kumagai and Lypson [10] argue that true fluency, not just competency, in multiculturalism requires critical self-reflection of one’s assumptions, biases, and values. Additionally, the authors add that critical self-reflection, along with cultural discourse “anchors a reflective self with others in social and societal interactions” (p. 783). Previous research has demonstrated the development and qualitative evaluation of courses for medical students [11] and medical faculty [12] that sought to reduce bias in medicine through lessons on introspection and cultural identity. However, as these studies have not reported follow-up data, there is little known of the course's lasting impact.

During the second section, we focus on interpersonal awareness. The aim is for participants to deepen their understanding of key terms (i.e. bias, stereotypes) and explore how cultural identities can affect interactions between mentors and mentees. Critical race theory (CRT) [13] informed the development of these “interpersonal” activities and includes an emphasis on institutional racism within US society and the intersection between race and power. By refuting tenets of colorblind ideology and integrating historical analyses, the CRT approach encourages behaviors that *eliminate* (not just document) racial equities [14]. We believe that the interpersonal awareness activities lead to more culturally aware mentoring behaviors.

Finally, section three focuses on skill-building. In this section, we use case studies and role-playing exercises to introduce racial/ethnic issues in a mentoring context, and encourage participants to build and practice critical thinking and communication skills. According to the Transtheoretical Model [15], the processes of behavioral change include self-efficacy, weighing the pros and cons of new behaviors (“decisional balance”), and contingency planning. The transtheoretical model has been a framework for assessing the impact of a diversity training among academic mentors [16]. We believe that role playing and case study activities give the participants an opportunity to discuss, practice, and observe culturally aware behavior, consequently building their self-efficacy to perform these actions with mentees or colleagues in real time.

We have previously reported evidence from a pilot study that CAM interventions increased research mentors’ knowledge of cultural diversity factors and their implications for research training of diverse trainees, their willingness to broach topics of racial/ethnic diversity, and their understanding of how their personal racial/ethnic identities impact their research mentoring relationships [5]. Co-facilitators of CAM are purposefully paired such that they vary across demographics, including race and ethnicity, career stage, disciplinary training, and gender identities. This is done so that the participants can hear different perspectives throughout the training, and so that they may feel a level of relatability and trust in the facilitators. Byars-Winston and colleagues [5] noted that CAM facilitators were alert to reading the “emotional tenor” of the participants and that across the three implementations reported on here, the CAM facilitators were rated highly. The ‘culture box’ activity was a part of the intrapersonal section, but due to its placement on the schedule and the content discussed, it also served as an effective approach in getting participants to open up and engage in authentic exchanges with one another during the training. This activity and the non-judgmental, engaging demeanors of the facilitators created an environment that supported collective learning.

The data in this paper report follow-up interviews from a sample of participants in the pilot CAM study [15]. We acknowledge that faculty mentors engage with CAM from different starting points and, therefore, we expected that participants would differ in their reported outcomes and impact. We were guided by the following research questions: 1) What changes in attitudes, norms, skills and behaviors occurred as a result of participation in the CAM intervention? 2) What components of CAM are associated with changes in individual participants? 3) What factors help participants to make change after the CAM intervention? 4) What barriers or challenges do participants encounter after the CAM intervention? The findings from this study are significant in providing support for the value of implementing the CAM curriculum and in describing what outcomes and impact might result from this intervention.

## Materials and methods

Data for this study were collected from participants in CAM interventions delivered at three institutions: 1) a private, minority serving institution in the south; 2) a consortium of state universities in the west, with a significant focus on inclusion of administrators and program directors of mentoring and/or diversity programs; and 3) a west coast large urban, minority serving state university. All participants from the three pilot cohorts were invited by email to participate in a follow-up interview 18–24 months after the intervention.

### Data collection

The interview protocol (See S1 Appendix) addressed: memorable activities from the intervention; emotions and thoughts during the intervention; personal, interpersonal, or institutional changes that they have attempted since the intervention; facilitators to changes and barriers to the activation of such changes after CAM; and recommendations for future CAM sessions. The data collection and consent procedures used in this study were reviewed and deemed exempt by the Health Sciences Institutional Review Board at the University of Wisconsin, Madison #2016–0458.

Prior to the interviews, participants received informed consent documents via email. Two CAM team members (VW and SH) and a third individual from a related research project conducted all interviews by telephone. Two interviewers are white women and one is an African American woman. While one of the interviewers (VW) had been a training facilitator at one of the three sites, she did not conduct any interviews at that site; no participants were interviewed by the person who conducted their training. The interviews ranged from 18 to 45 minutes. All interviews were audio recorded and professionally transcribed verbatim. Names and identifying information were omitted from the interview transcripts.

### Data analysis

The qualitative analysis software NVivo was used to facilitate data coding and analysis. We used an inductive and iterative approach using “constant comparison” to identify themes in the interviews and compare our interpretations across participants [17]. Each interview transcript was first annotated independently by two researchers (RM, VW), the results of which were compared in order to generate a descriptive coding architecture within NVIVO. This architecture was reviewed by the larger study team (RM, VW, SH, SCQ, ST, CW) and adjustments were made before the team conducted an analysis and collectively identified themes. These themes included: what activities participants remembered from CAM and the extent they associated those activities with change; what changes they reported in attitudes, norms, skills and behaviors; what facilitators and barriers to change they experienced after CAM; and finally, what recommendations they had for improving CAM. The final framework of intrapersonal, interpersonal, and behavioral change used here to describe workshop impacts, parallels the three components of the CAM intervention: intrapersonal, interpersonal and skill-building.

### Sample

All participants from the three trainings (n = 74) were contacted and 24 (32.4%) agreed to participate in interviews. Of the 24, six were from the large public university and nine each were from the other two sites. Eighteen of the 24 are women. The demographic information of the full group of CAM participants have been previously described [15]. As these data were not individually identifiable, correlations from the earlier study could not be made to participants interviewed here; demographics were not collected as part of the current study. Some of the interviewees indicated their race or ethnic background during the interview. Based on this volunteered information, we know the sample included African American, Hispanic, White and Asian men and women.

## Results

We report findings from our interview data illustrating how three observed impacts of the CAM intervention—changes in intrapersonal awareness, interpersonal relationships, and skills-based behaviors—work in concert to facilitate participants’ pathways to becoming culturally aware mentors. We describe the aspects of the training that participants best remembered as having impacted their personal growth, interactions with others, and efforts to stimulate cultural change in their environments. We begin by describing the activities that participants found most memorable and impactful and continue to describe how the training has gone on to impact their lives at their home institutions.

### Reflecting on activities: What participants remembered

We asked each of the participants what they remembered from the CAM training—that is, what “stuck”- one to two years after participation. Table 1 notes the number of participants who recalled specific activities within the day-long workshop.

**Table 1:** Activities in CAM training recalled by participants.

Activities	Section	% of people (n = 24)
Culture Box	Intrapersonal	54.2% (13)
Role Play	Skill building	37.5% (9)
General Discussion	All three	33.3% (8)
“A Tale of O” Video	Interpersonal	25.0% (6)
Discussion of Biases	Interpersonal	12.5% (3)
Case Studies	Skill building	12.5% (3)

The activities mentioned most frequently were those that were seen as being most novel or that elicited an emotional response. These included the culture box, the role play, and the video “A Tale of O.”

### The culture box

In the culture box activity, participants were asked to bring in two or three items that represent their cultural identity. This was the opening activity of the workshop and was designed to stimulate participants’ reflection of themselves as cultural beings and to set the stage for open discussions. The culture box was described as “powerful;” a couple of participants noted that it made them feel “vulnerable.” One relayed that it “makes you vulnerable [to] different groups, in terms of exposing you to others who may have completely different experiences than you.” Another participant connected vulnerability to building interpersonal connections, noting “It was…good [to] step outside of your comfort zone, [and] then eventually be able to learn about and understand each other at [an] even more personal level.” Most participants who remembered this activity described it as having facilitated open communication and connection in their groups. They appreciated getting to know their colleagues on a more personal level, or learning about people beyond “the formal job title.” One noted,

“…I think that that was a good grounding exercise for many people to kind of come into themselves and their identity and be centered in that experience…”

Participants also noted how this activity impacted their cultural awareness. For example,

‘…I really appreciated that exercise, as a way of realizing that everyone has their own, their own story…. What really moved me is to realize that everyone has this background, that people grow up in a cultural context…”

“…I thought that was so beneficial and so useful to really expand upon our own life experience because, you know, when we, when we profile each other like unconsciously and just look around, you know, like we don't really know the complete backgrounds of what a person's gone through,…that project really shed light on all of our unique histories…

The latter participant was one of two who mentioned that they went on to implement the activity with their own mentees as a way to broach topics on identity. She said, “It starts conversations about some aspects of identity that we don't always openly talk about.”

### Role play

Another activity that participants remembered frequently was the role play, in which they had the opportunity to respond to case scenarios and practice having conversations with mentees around sensitive topics. One participant appreciated hearing the strategies that their colleagues employed:

Listening to the…subtleties of the interactions and…how things don't have to be totally in your face, to be, maybe, disruptive or positive. But the…subtleties in the communication that can be related to not being aware…of cultural differences or cultural expectations.

One of the things that I—I remember still thinking about is, again, those really awkward role playing conversations. And I think one of the things that I've been thinking about since then is not just the awkwardness, but being like sort of learning from how different people handled that in the room. And—and I haven't been in any of those types of roles, but I just remember thinking like, oh, that person, you know, had—had a really good way of talking about this when things got sort of tense during the fake conversation. And oh, that person brought up something that I didn't know anything about before as they were trying to explain what was going on in this role playing. So, those are some of the things that just keep coming back to me. ….[the] awkwardness…was really beneficial

Another reflected on how participants used the role play to share their own experiences:

During the role playing especially, it seemed to me that a lot of people, including myself, kind of used that as a way for our own experiences to come out…So, a lot of people were bringing what had actually happened to them as underrepresented minority students…to these—to the sort of artificial conversations. But it was really clear that for some of the speakers, it was coming right from their own heart because they knew exactly what this role was because they had lived it before.

### A Tale of O

Aside from the general discussion with peers, another activity that left an impression on participants was the video, “A Tale of O” that highlighted what it is like to be the only visual member of a specific group. Participants noted the video made it easy to connect to the idea of being discriminated against and how it makes one feel. For example, one notes,

It helps to think about other types of diversity or other—for me, that was the key thing there was just like oh, I felt like it represented it, multiple types of diversity, multiple types of ways that people could be discriminated against or stereotyped negatively. It's a non-threatening way because it was acting as an O, so I felt like whatever, whichever way that you've been discriminated against, you could relate to it through that video. I searched, trying to find that video again to use it myself. So, if it is publicly available, I would love to have that…I would use it for training within my own team. And probably also would be using it in the classroom.

Another mentioned that it was “just something I really connected with,” and mentioned that s/he would have liked to share it with colleagues.

Participants reflected most often on the Culture Box, role playing, and the Tale of O video, reporting that these activities helped to break down relational barriers within the group and began to facilitate participants’ understanding of themselves and others across cultural groups. Many also noted that the activities brought a chance, whether by design or not, to bring one’s own experiences to the forefront and reflect on those or make them visible to the group. Finally, participants found these activities accessible in the sense that they saw them as potential activities that they would use with their own mentees.

### Lasting impacts of CAM

CAM participants reflected on the training in a variety of ways, noting the effects it had on their perceptions of and experiences in their home institutions. While the main focus of this discussion is based on changes in intrapersonal, interpersonal, and behavioral processes in mentoring, interviewees discussed other concrete takeaways that can be captured under more general themes. Most significantly, participants discussed shifts in their own relationships with their faculty peers and reflected on what constitutes a good facilitator in a mentoring training program.

### Impacts on peer relationships

One of the most discussed benefits of the training was the impact it had on participants’ relationships with their own peers–other faculty members and administrators in their institutions. Ten participants we interviewed discussed their peer relationships at length and elaborated on how the tools they picked up from the training has helped them in their collegial relationships. Half of the participants commented on the credibility and expertise of the facilitators, which inspired trust and perceived legitimacy in the curriculum. Several participants stated that the facilitators were effective in making topics concrete that are often elusive and hard to articulate. For three participants, most significantly, the training helped them become mentors to their own peers, helping their fellow faculty members manage challenging situations with students or improving their own skills in leading diversity programs in their own institutions.

One participant discussed the impact of the training among a group of faculty members who attended the training together, stating,

I think it helped the most with my [campus diversity program] where we communicate a lot better than we had in the beginning. I think there's more openness in our communication, a lot more listening of people's different experiences definitely than before. So I would say that's probably the biggest change.

Another became better attuned to other faculty members’ discomfort in interacting with HU students:

They're very uncomfortable having the conversation with their minority students. They pretend it away and don't address it… You have to want your students to excel. You have to be honest about some of the challenges that they may face. And I don't think a lot of my colleagues are honest about things, and I don't think that they're willing to face those things.

This same participant also discussed ways of advising peers around mentoring relationships with students, and the challenges posed by helping to educate peers:

They want their students to feel comfortable coming to them. One in particular, she said to me that she had a very good mentoring relationship and she's from a different ethnic group. She had a very good interaction with her mentor and continues to reach out to her mentor and they have a great relationship. She had hoped to have a similar relationship with her student and that had not been the case. And her question is, ‘What am I missing or what am I doing wrong?’ Those types of things. And I gave her some suggestions. I don't know if she was willing to make the same assessment or it was more work [than] she was willing to do.

Discussions about perceived barriers to promoting culturally aware mentoring came up in the interviews, as the previous excerpt displays. Interviewees were often candid about the challenges posed in promoting cultural awareness on their campuses. Despite leaving the training with new tools, the realities they encounter at their institutions are inherently challenging for non-white students and faculty. One participant stated:

One of the things that's really hard is to create spaces where non-white faculty are not going to be harmed by conversations about race or bias or structural inequity, because our institution is so majority white. If we try to have a conversation about students who feel that there's bias in the assessment process, then white faculty quickly get very defensive about it. And then that creates a dynamic that is not helpful for the faculty of color in the room…

### Intrapersonal, interpersonal, and behavioral based changes

As noted, the training is organized around three sections, intrapersonal awareness, interpersonal awareness and interactions, and skill-building to facilitate behavioral change. The majority of interviewees reported the CAM workshop had one or more impacts on their intrapersonal awareness, interactions with mentees, and mentoring behaviors. Here, we provide examples of how the training affected participants in each area, as well as noting how they work in concert.

Notably, the interviews did not in any way ask participants to reflect along the three workshop outcome elements of intrapersonal awareness, interpersonal shifts, and behavioral change. Rather, alignment with these themes came from listening to how participants described the various impressions and impacts of the training, up to two years after the training. The quotations featured represent the three themes, but are far from exhaustive.

### Intrapersonal

Many participants discussed how the training increased their cultural awareness, which often led to feelings of increased confidence and deeper understanding of cultural differences. Some remarked on the emotional impact the training had on them and their willingness to open up around their colleagues. In some cases, the intrapersonal work also led to shifts in interpersonal engagement or motivation to change their behaviors. For some participants, the intrapersonal realizations had the most profound impacts. Two participants noted:

It's really made me, I think, more reflective, or introspective maybe is a better word, about my own sort of view point of things, my own biases, my own prejudices, really making the effort to sort of discern those things and figure out what's going on inside of me, especially in my relationships with others.

It was nice to sort of reflect on, you know, my own awareness of my race and my social category. I kind of get so caught up in thinking about other people that I don't always [consider] my own, and so especially because I consider my background pretty boring. You know, so really kind of reflecting on that and trying to see where that gap might be and how to fill it.

One participant noted that sharing experiences of her personal background with her colleagues during the “culture box” exercise provided a better understanding of her colleagues and herself. She noted that the exercise facilitated, “understanding at a deeper level, at a more personal level, which I really appreciated. And sharing for myself, it was interesting, because it is kind of a vulnerable experience to be able to share about your personal background.” Though she still felt she had more to learn, she felt that the training “did help me have an increased awareness and understanding about how those differences can be very real and can potentially impact students.” Ultimately, this participant described how gaining cultural awareness might increase her awareness of what her students might be facing.

For many, these intrapersonal realizations, where participants gained a better understanding of their own attitudes and competencies, fed into shifts in their attitudes and reactions toward others. As the following discussion demonstrates, rarely does a realization of one’s own attitudes and biases remain confined to the intrapersonal.

### Interpersonal

Many participants described gaining awareness that impacted their relationships and interactions with others. These “interpersonal” revelations set some participants up to behave differently towards their students and mentees. For example, one participant realized that her perspective on “colorblind” racial ideology made her inattentive to cultural differences. This led to a change in how she related to others. She said,

I think [the training] has opened my mind into understanding that there are differences…I always looked at myself as someone who is colorblind and culture-blind in the sense that I didn't judge people based on their color and culture. But there is another side to that, as well. Other than not judging people on it, you have to show your respect to it. And there are times that you need to acknowledge that difference. And so that's what I learned from these workshops, that just not judging people based on them is not enough. Sometimes you do need to acknowledge the difference and validate the difference because the difference is a good thing, it's not a bad thing.

Another participant remarked that the culture box exercise during the training showed him how to respect others’ differences and be more receptive to the personal dimensions of his students’ lives. The increased awareness to differences allowed him to make fewer assumptions about students and to listen to them more closely. He noted,

A lot of our students are first generation, they may have different economic backgrounds, they may have different family circumstances…The training, I think, helped us understand. We need to listen a little bit more to hear other people's experiences, to help formulate, you know, what the next steps are when we're working with students.

He noted that the training helped him “check in” more often with students and be more personally open with them, though this participant was at a nascent stage of enacting concrete changes. Again, the increased interpersonal awareness tended to lead participants towards actual or planned behavioral shifts. That shift may be subtle, as in the above participants’ pledge to listen to his students, or, it could be more profound, as in the concrete behavioral changes categorized below.

### Skills and behaviors

A number of participants gave examples of acquired skills that led to changes in their behaviors with students and in their institutions. They found these skills useful in teaching and mentoring situations. For these participants, increased comfort and competence in recognizing cultural insensitivities led to a change in their interactions with students.

One participant described changes in his interactions with students in the classroom. He noted that the training helped him become more comfortable intervening in student discussions when he noticed culturally insensitive comments arising. He said that the training gave him “more confidence in sensing when a discussion is about to go off-topic or go in a way that could really affect someone…to say, ‘no, we're going to stop this before this word comes out of their mouth.’” The training helped him recognize potentially insensitive comments, but, more importantly, it gave him the confidence to intervene constructively. He expanded,

[I am] more comfortable navigating discussions that are needed about cultural competency and things like that…more comfortable speaking up when someone says something that I'm like, ‘Oh my goodness, what did you just say?’ I think particularly with students, I'm more comfortable saying, ‘OK, time out.’ So, I think all of the trainings have helped me gain a little bit more confidence in my ability to speak up.

In some cases, narration of behavioral change reflected a broader shift in one’s attitudes, perceptions, and approaches to mentoring. The cases below reflect how the three components work together for more comprehensive shifts in participants’ experiences with culturally aware mentoring.

### Case examples of intrapersonal, interpersonal, and behavioral change

A small number of participants narrated a profound change impacting their intrapersonal awareness, their interpersonal interactions, and their skills and behaviors in mentoring situations. During interviews, these participants described the ways the training had an impact on their competencies, their perceptions of others, and their motivation or actualization toward behavioral change. The three cases below provide evidence that the CAM training, though focused on race and ethnicity, gave participants awareness and confidence to recognize and address other dimensions of cultural diversity as well as inequalities that occur in their training environments. Collectively, the cases also illustrate participants’ awareness of and willingness to address matters of inclusion in their mentoring and training environments.

*Case #1*. This participant told the interviewer that she entered the training with skepticism, because she had not found diversity trainings to be very useful in the past. In this training, however, she noted an increase in her “cultural awareness,” which ultimately led to changes in her interactions with students and mentees. She noted that the first thing that she noticed in the training was that the facilitators were “effective in explaining the delicacy in culture and they also mentioned the limitation that one person from one culture can [have in trying to] understand the other culture.”

She noted in particular that the training helped her learn to recognize her own “privilege” and be more aware of her surroundings, and that intention does not always equate to impact, indications of intrapersonal change. She said,

I am more mindful about my surroundings and more mindful about my mentees and my students. I try to be careful…My privilege may influence other people, because I am talking to my mentee or students. So sometimes, I may say something that I didn't really mean to say, or my intention can be perceived incorrectly from what I intended. So, I'm mindful about that.

This participant self-identified as female and Asian, and referred to her privilege with regard to her students who are from historically underrepresented backgrounds.

In addition to her heightened awareness, the role-playing activity helped her understand how she might react to difficult situations with students, aiding in her interpersonal understanding and preparing her for real behavioral change. She noted that as a statistics professor, she does not have many opportunities to engage with students on cultural matters, and so the training helped prepare her for situations that could arise. She reflected,

I mainly teach statistics, so I usually am not dealing with those culture-related materials in my class. Even though I am [Asian,] I have not really paid attention to that kind of a subject…The workshop really opened my eyes…I have to think about the different cultures. I realized that my insensitivity to different cultures [affects] my role as teacher. Sometimes, I make unintentional remark to students. I am more attuned in my own behavior as I'm actually teaching my students…I have one mentee who [made] me reflective of…not having much of a clue about my surrounding.

She then offered an example of a substantive change in her interaction with a mentee. She described a mentee who self-identified as a gay man and explained how the interpersonal skills from the training helped her connect better with the student and create a more open environment for him. She said,

My student is gay, and he was having relationship issues. Before, I wouldn't have known how to approach him, but I felt more comfortable…and I made him feel comfortable [opening] up to me…From the bigger perspective, everybody goes through relationship issues. Also, I was able to point out someone whom I know [to be] very open about [these] kind of issues…I told him that each of us has our own background…but you can understand that person and where that person's behavior [is] coming from. And that person's identity…just by understanding that, we can feel comfortable about it. That's how I handled it.

The participant is clear that her newfound understanding of cultural identity helped her navigate a situation with a mentee in a way she would not have been able to before the training. She described how her newfound comfort in relating to students despite their differences helped her provide resources for the student and offer an open environment to discuss his problems.

*Case #2*. This participant, who noted that she ran the mentoring training at her own institution, said the CAM training had two main effects: it made her more reflective of her own biases and prejudices and it gave her insight on how to tailor mentor trainings at her own institution. Both shifts were brought on by intrapersonal reflections resulting from the training, which led to shifts in perceptions of and interactions with students, as well as concrete changes in the mentor training at her institution.

The training activities heightened her awareness of her own biases, which she credited with making her both more introspective about herself and more effective at interacting with colleagues and mentees. Referring to the culture box activity and subsequent discussions, she noted:

You had to bring an item that reflected who you are as a person…And at some point during the workshop, you know, you talked to others in your group about why you brought that item and why it represents you and why it's so important to you…For me personally, what I've really started to question about myself was how much I recognized my own biases and my own prejudices. I have these things, am I really seeing myself the way I need to, especially, you know, when it comes to interacting with others and doing what I need to do. It made me more introspective and more cautious about what I was bringing to a relationship with a mentee or with a colleague for example.

The experience of identifying her own cultural object, reflecting on it, and discussing it with others helped her become more introspective, and she gained an awareness of her biases and attitudes towards others. She credits this with an increased sense of “caution” in mentoring situations, an interpersonal effect. When probed by the interviewer, she elaborated more carefully on how this newfound sense of herself has impacted her interactions and caused her to change her behavior towards students. She said,

I always felt a sense of comfort in dealing with the students [at my university], because we share a racial background. But what I came to realize, is that we don't necessarily share a consistent background…I began to think about what I was bringing as far as a perspective and it came up with me with the [Orlando] Pulse Nightclub shooting. I'm heterosexual. I personally do not believe in any kind of discrimination against anybody, so I have nothing against homosexuals, anything. But before that training, I don't know that I would have ever done what I did after the shooting. We had a [mentoring session] with our students, and I remember going in and I spoke to the students about the shooting and I cautioned them against blowing it off because maybe they weren't gay or maybe nobody close to them was gay…I cautioned them to be vigilant about taking care of one another, whomever they are, and to stand up for whomever is being wronged. I don't think I would have had that kind of cerebral process had I not gone to that mentor training.

This participant reflected on the comfort she felt as a faculty member at a minority-serving institution and challenged herself to reflect on her own biases and create a caring environment for all. She reflected again on changes later in the interview, when she elaborated on how she adapted components of the program to improve mentor training on her own campus. She said,

Right after [the training], and I remember this very well, I was really focused on our own

mentor training and what we needed to do to address [biases] as a whole…I was truly focused on, all right, this is sort of a foundation, how do we build on this in our own. We had the infrastructure in place, meaning we had the mentor training program in place, and part of that mentor training program was [about], I won’t say cultural awareness, I’ll say, stereotypes…that was already a part of it. And so because that infrastructure was already in place, I had something to work with. I also had a faculty member who's researched in this area and could bring it to an audience in a non-threatening, empirically based way. And I think all of those things worked together so it's just not some person standing in front of you wagging their fingers saying you need to do better. All of that worked together to make that possible.

*Case #3*. The CAM training had an impact that represented all three CAM training components on at least one participant with more extensive training in mentoring leadership. Rather than describe the training as providing initial exposure to cultural awareness, she reported that it inspired confidence and reinforcement in her process of becoming a mentoring leader on her campus. It also expanded her vocabulary around mentoring practices. She stated,

You know, in some ways, it empowered me. So, it reinforced a certain kind of

awareness that I already had, but…also I could recognize myself as part of a community that was dedicated to addressing these issues. I felt stronger in my position on my campus in reaching out. And also, using the language of the workshop and just using the phrase Culturally Aware Mentoring is actually a really helpful phrase to use.

1. Her increased confidence enabled her to provide support for HU students who were not receiving adequate mentoring, and to better identify situations when students are receiving poor mentoring. She described when a student of color approached her to report a poor experience with a mentor. She described the situation:

[A student said he’d] been told [he has] to do this presentation and [he didn’t]t really understand the presentation. And I was like, ‘Did you ask your mentor for more information?’ I try to also coach them on ‘mentoring up’ and being proactive. And he was like, ‘Yeah, but [the professor is] hardly around and he told me there's some information on our group drive I should just read…but I couldn't find it.’ I [reached] out to his mentor. And the mentor pretty much made it clear to me that he didn't have time to [give] the student close guidance.

By recognizing these types of situations, she began to think about how to improve mentoring of recently recruited HU students. She attributed her ability to intervene in mentoring relationships to the confidence inspired by the CAM training. She said:

I don't know if I would have had the confidence to address an issue that came up recently with one of my diversity students who had been placed in a research group and wasn't getting proper mentoring. This group had been very energetic in trying to recruit underrepresented students…but when I found out [what] their actual practices were, I could see big flaws. And so, I was able to talk to the PI that ran that group…I was able to really engage him and talk about this question of, ‘What kind of a culture do you see yourself creating?’ And to talk about being culturally aware in terms of what are the specific cultures of our students, of their personal histories, or their family culture, or their [identity] in terms of race or ethnicity, and thinking about those cultures…to be able to frame things in terms of the culture of a working group or lab, and in talking about how that culture is or isn't inclusive.

Her intervention into her colleague’s practices was fruitful. It spurred interest in a mentor training for his research department, and allowed them to leverage campus resources for mentoring training. She reported,

I did get the PI interested in doing a mentor training for his staff researchers and graduate students, and also to bring in our campus office of conflict resolution that does implicit bias training. And we're going to be scheduling that [in] the next month or so,.

Altogether, these examples represent the impact of CAM training on a diversity of participants ranging from the skeptical and novice to the experienced and converted, and from those who are working directly with trainees to those who are working on institutional efforts to advance effective mentoring.

## Discussion

The goal of this paper was to describe the impact of CAM training on participants 18–24 months after training. Interviews with participants showed that the curricular design was effective in drawing out open, honest self-reflection about cultural diversity and deep, empathic learning between participants [5]. Participants also reported the positive impact of trust that was built when the facilitators demonstrated their skills to deliver the content effectively and with clear command of the program materials. The combination of curriculum design and trust in expert facilitators resulted in a lasting experience for many of these participants. Further, this combination facilitated participant introspection, which was effective in prompting participants to examine their own beliefs and behaviors around racial and ethnic diversity in their mentoring relationships and in their institutions. Introspection, which participants often reflected on as “vulnerability,” allowed trainees to consider their interactions with peers and to think through and ultimately act on new insights. The interpersonal aspect of sharing these insights within a community promoted the intrapersonal and behavioral changes captured in the data. Our data confirmed that introspection and awareness of cultural differences are an essential first-step in encouraging faculty behavioral change to promote inclusion and belonging in STEM research training contexts.

Our findings revealed that the CAM training impacted a wide variety of participants. Some were new to mentor training and early in their academic careers. Others were very much “in the trenches” and working directly with students and administrators responsible for institutional mentoring programming. Some self-identified as majority white, and others as from historically underrepresented groups. Though the impacts of the training were different across groups, participants with varied levels of experience and access benefited from the training in profound ways.

Remarkably, our interviews provided ample evidence that CAM training promotes behavioral change not only in mentoring, but also in participants’ relationships with colleagues and administrators. Multiple respondents reported making additional efforts with colleagues to promote mentor training in their institutions, with some reporting heightened confidence to intervene with faculty peers who were not providing good mentoring to HU students. This latter point is particularly notable given the importance of addressing inclusion for all students, something that mentors are well-positioned to do but may not regularly think to do. Our data suggest that CAM training can lead to lasting changes in the beliefs and behaviors of faculty and administrators related to cultural awareness in their own mentoring with students as well as their attention to mentoring efforts at the institutional level in their home campuses.

The results of our interview data from CAM participants make clear that mentor training targeted to cultural awareness through the entry point of personal cultural self-awareness and introspection, coupled with sharing these insights in community, can be effective in prompting changes. Our findings are consistent with neuroscience research by numerous scholars [18]. that self-awareness is the key life skill upon which other life skills like self-mastery and social skills essential to successful human interactions are based. Siegel [19] summarized neuroscience discoveries illustrating that self-reflection and personal insights stimulate the same circuits in the brain that create well-being and underlie compassion and sympathy. In alignment with this observation, participants’ cultural self-awareness facilitated through the CAM training may increase their ability to have empathy toward their HU students and their attention to cultural dynamics in their mentoring relationships. Our results are promising and provide a foundation for future investigation. For example, a future CAM study could help identify, with greater confidence, what factors in CAM training, either individually or in combination, account for the observed changes in participants’ beliefs and behaviors toward culturally aware mentoring. This type of investigation would allow for testing hypotheses and models of predictive relationships among theoretical constructs that explain the mechanisms through which the CAM training curriculum has its effect on participants and their mentoring practices.

The CAM training is one model for promoting more effective mentoring practices and more attention to institutional mentoring efforts by faculty members and administrators who are responsive to efforts to diversify and inclusion of the STEM workforce. The future academic and career success of HU trainees will likely be improved by senior faculty and administrators who are equipped to facilitate their talent development through culturally aware mentoring.

### Limitations

Our interpretation of CAM’s long-term impact is limited by the high percentage (67.5%) of training participants who were not interviewed for this study. The lack of response could indicate lack of remembering or interest, being too busy, or they may not have been deeply impacted by the training. Additionally, those who participated in this study may have been motivated to respond because they had positive updates to share. Therefore, it is important to note the potential non-response bias that may be informing our findings. Further, we did not analyze the data by demographic subgroups, given the small sample size that would preclude drawing inferences from the data in this way.

### Future directions

With the benefit of future funding, the CAM team and investigators will conduct a national study with targeted dissemination of CAM training to investigate its effectiveness in a variety of graduate research training contexts, as well as the degree of effect achieved with variations of the original training design.

## Supporting information

S1 Appendix (DOCX)
