# Supplementary material for: Culturally aware mentorship: Lasting impacts of a novel intervention on academic administrators and faculty
Source: PLoS One. 2020 Aug 7;15(8):e0236983. doi: 10.1371/journal.pone.0236983 (PMC7413486; doi:10.1371/journal.pone.0236983)
Supplement: S1 Appendix — (DOCX) [file pone.0236983.s001.docx]

**Appendix A:**

**Interview Questions**

Thank you very much for agreeing to reflect on the time since you participated in the Culturally Aware Mentoring (CAM) Workshop. We are interested to learn more about if and how people use what they learn from the workshop. We are going to start by asking you some questions about what you remember about the workshop, and then ask you to reflect on how your experiences in the workshop have impacted your mentoring and your relationships with others. We’ll close with asking for any suggestions you might have to help us improve the workshop.

1. What do you remember about the CAM workshop?

2.    Now several months after you experienced the CAM workshop, any reflections on how it most impacted you?

3.    Since you attended CAM training, have you had new thoughts or realizations about anything you learned during the workshop? How have these new thoughts or realizations changed what you do, particularly related to mentoring?

4.    Reflect back, best you can, to the time right after the workshop ended. What did you think that you were going to do with what you had learned that day?

5.    Since that time, did you end up doing any of the things you thought you might? Why or

why not?

6.    Do you think the workshop led to any changes in your mentoring or other interactions with students/trainees (UG student, GS, postdocs, jr faculty)?  If so, in what ways? Have you seen changes in your mentee(s)?

7.    As of today, how comfortable or competent do you feel using the principles and practices of culturally aware mentoring within your mentoring relationships?

8.    Is there anything you learned in the CAM workshop that you continue to struggle with?  (internally/personally as well as in the environment) How so?

9.    Have you talked about the CAM workshop with anyone, like colleagues or anyone outside of your work environment?  What has been their response?

10.  Do you think the workshop led to any changes in your interactions with colleagues (those who went through the workshop and those that did not)?  If so, in what ways?

11.  How can the workshop be improved or be more effective?
